# Long-term outcome of stereotactic brachytherapy with temporary Iodine-125 seeds in patients with WHO grade II gliomas

**DOI:** 10.1186/s13014-020-01719-9

**Published:** 2020-12-09

**Authors:** Juliana Watson, Alexander Romagna, Hendrik Ballhausen, Maximilian Niyazi, Stefanie Lietke, Sebastian Siller, Claus Belka, Niklas Thon, Silke Birgit Nachbichler

**Affiliations:** 1 Department of Radiation Oncology, University Hospital, LMU Munich, Marchioninistr. 15, 81377 Munich, Germany; 2 Department of Neurosurgery, München Klinik Bogenhausen, Munich, Germany; 3 Department of Neurosurgery, University Hospital Salzburg, Paracelsus Medical University, Salzburg, Austria; 4 Department of Neurosurgery, University Hospital, LMU Munich, Munich, Germany; 5 German Cancer Consortium (DKTK), Munich, Germany

**Keywords:** Stereotactic brachytherapy, Iodine-125 seeds, Grade II glioma, Low-grade glioma

## Abstract

**Background:**

This long-term retrospective analysis aimed to investigate the outcome and toxicity profile of stereotactic brachytherapy (SBT) in selected low-grade gliomas WHO grade II (LGGII) in a large patient series.

**Methods:**

This analysis comprised 106 consecutive patients who received SBT with temporary Iodine-125 seeds for histologically verified LGGII at the University of Munich between March 1997 and July 2011. Investigation included clinical characteristics, technical aspects of SBT, the application of other treatments, outcome analyses including malignization rates, and prognostic factors with special focus on molecular biomarkers.

**Results:**

For the entire study population, the 5- and 10-years overall survival (OS) rates were 79% and 62%, respectively, with a median follow-up of 115.9 months. No prognostic factors could be identified. Interstitial radiotherapy was applied in 51 cases as first-line treatment with a median number of two seeds (range 1–5), and a median total implanted activity of 21.8 mCi (range 4.2–43.4). The reference dose average was 54.0 Gy. Five- and ten-years OS and progression-free survival rates after SBT were 72% and 43%, and 40% and 23%, respectively, with a median follow-up of 86.7 months. The procedure-related mortality rate was zero, although an overall complication rate of 16% was registered. Patients with complications had a significantly larger tumor volume (*p* = 0.029).

**Conclusion:**

SBT is a minimally invasive treatment modality with a favorable outcome and toxicity profile. It is both an alternative primary treatment method as well as an adjunct to open tumor resection in selected low-grade gliomas.

## Background

The management of low-grade gliomas WHO grade II (LGGII) is still a difficult undertaking. Several evidence-based clinical practice parameter guidelines have been formulated [1–6]. The results of the recently published EORTC 22033–26033 study further stress the importance of molecular analyses to individualize LGGII treatment [7].

Stereotactic brachytherapy (SBT) is a safe and effective local treatment option in selected LGGII. The steep dose gradients enable the delivery of a high dose to a defined target volume, while sparing surrounding healthy tissue [8]. Assessing the invasiveness associated with open brain surgery and the treatment-related side effects as well as the lifetime dose limits of external beam radiotherapy (EBRT), Iodine-125 brachytherapy is a suitable modality that leads to only a few complications in patients [9–11].

The primary objective of this retrospective analysis was to assess survival time and outcome of a large cohort of 106 consecutive patients with histologically verified LGGII treated with SBT with low-activity temporary Iodine-125 seeds. Prognostic factors were analyzed in respect to their relevance on survival time after seed-implantation. Furthermore, the influence of additional applied treatment was considered.

## Methods

### Patient selection and inclusion criteria

This single center analysis included all consecutive patients with LGGII who received SBT with temporary Iodine-125 seeds at the University of Munich between March 1997 and July 2011. Treatment in favor of SBT always was recommended in consensus by the interdisciplinary tumor board according to the in-house standard operating procedures for LGGII: In here, SBT was recommended for newly diagnosed or recurrent, unifocal, circumscribed, virtually spherically shaped, histologically verified astrocytomas, oligoastrocytomas or oligodendrogliomas WHO grade II with a size not larger than 4 cm in neuroimaging data, which were not deemed appropriate candidates for complete safe resection [8]. In individual cases, patients may have become candidates for SBT because they declined the recommended open brain surgery. Moreover, in larger, partially eloquent LGGII SBT may be combined with calculated incomplete resection as a risk-adapted, combined local treatment option [12]. Generally, treatment was indicated in patients presenting with new clinical signs, exacerbation of previous symptoms and/or neuroradiological findings of tumor progression.

Exclusion criteria were: any histology other than astrocytoma/oligoastrocytoma/oligodendroglioma WHO grade II; suitable for complete safe resection; not well circumscribed tumor, multifocal tumors, tumor larger than 4 cm in neuroimaging data.

### Patient evaluation

By reviewing medical records of each patient, data about patient characteristics (Table 1), complications (Table 2), the chronological course of the disease, and different treatment modalities that were used before and/or after SBT (Table 3) were acquired. Furthermore, the acquired information included histological classification, potential malignant progression and molecular biomarkers that have been routinely determined in our institution since mid of 2004 for loss of heterozygosity (LOH) on chromosome 1p and /or 19q, mid of 2007 for O-6-methylguanine-DNA methyltransferase (MGMT) methylation status, and mid of 2008 for isocitrat-dehydrogenase (IDH) 1/2 mutational status (Table 1).

**Table 1:** Characteristics including histopathology, molecular biomarkers and dosimetry for all patients and the group of patients who had no therapy before SBT (SBT as first-line therapy)

Characteristic	Total number	SBT as first-line therapy
**Total number of patients**	106 (100%)	51 (100%)
**Sex**		
Female	47 (44%)	23 (45%)
Male	59 (56%)	28 (55%)
**Age at diagnosis in years (median)**	38.2 (range 1.0–70.59)	41.0 (range 1.0–70.5)
**Age at first SBT (median)**	40.5 (range 1.1–70.6)	41.2 (range 1.1–70.6)
**Tumor volume at SBT in ml (median)**	8.5 (range 0.4–50.5)	9.0 (range 1.3–34.7)
Number of patients < 20 ml	80 (75%)	36 (71%)
**Histology**		
Astrocytoma II	90 (85%)	50 (98%)
Oliogoastrocytoma II	8 (8%)	1 (2%)
Oligodendroglioma II	8 (8%)	0 (0%)
**Malignant transformation**		
Total count	46 (43%)	18 (35%)
Delay after diagnosis (median years)	7.5 (range 0.9–22.0)	4.0 (range 0.9–13.4)
Delay after 1st seed (median years)	4.4 (range 0.8–16.4)	3.8 (range 0.8–13.3)
**Molecular biomarkers**		
MGMT methylated	42 (40%)	12 (24%)
MGMT not methylated	9 (8%)	8 (16%)
MGMT status unknown	55 (52%)	31 (61%)
LOH 1p	2 (2%)	0 (0%)
LOH 19q	6 (6%)	1 (2%)
LOH 1p/19q	12 (11%)	2 (4%)
No LOH	27 (25%)	16 (31%)
LOH status unknown	59 (56%)	32 (63%)
IDH1 mutation	24 (23%)	6 (12%)
IDH2 mutation	0 (0%)	0 (0%)
No IDH mutation	2 (2%)	2 (4%)
IDH1/2 status unknown	80 (75%)	43 (84%)
**Dosimetry**		
Mean number of implanted seeds/patient	2.3 (range 1–5)	2.5 (range 1–5)
Median total implanted activity in mCi	21.8 (range 4.2–43.4)	21.8 (range 5.3–42.0)
Median reference dose in Gy	54.0 (range 40–60)	54.0 (range 50–60)
Median minimum tumor dose in Gy	30.2 (range 9.2–63.7)	30.2 (range 9.7–49.7)
Median maximum tumor dose in Gy	808.9 (range 235.5–2166.6)	771.6 (range 235.5–1848.3)

SBT, stereotactic brachytherapy; MGMT, o-6methylguanine-DNA methyltransferase; LOH, loss of heterozygosity; IDH, isocitrate-dehydrogenase

**Table 2:** Complications

Complication	No	Comments
**Periprocedural complications**		
Local cerebral bleeding	2/106 (2%)	
Seed repositioning due to migration and/or positional deviation from the original treatment plan	3/106 (3%)	
Wound healing disturbance with concomitant cerebritis whereupon a premature seed-explantation had to be performed	1/106 (1%)	
**Long-term complications**		
Prolonged edema	11/106 (10%)	Of the eleven, three developed radionecrosis, resulting in parenchymal bleeding with brainstem compression or hemiparesis
**Total**	17/106 (16%)

**Table 3:** Overview of the different treatment modalities including therapies before and after SBT

Treatment modalities in addition to SBT	Prior to SBT	Salvage therapy after SBT
No therapy	51	36
Resection only	43	5
EBRT only	0	6
Chemotherapy only	2	8
Additional SBT only	0	4
Resection + EBRT	4	0
Resection + chemotherapy	4	1
Resection + SBT	0	0
EBRT + chemotherapy	1	16
EBRT + chemotherapy	0	1
Chemotherapy + SBT	0	10
Resection + EBRT + chemotherapy	1	6
Resection + chemotherapy + SBT	0	0
Resection + EBRT + SBT	0	0
EBRT + chemotherapy + SBT	0	10
Resection + EBRT + chemotherapy + SBT	0	3
**Equals number of patients**	106	106

If a treatment was performed repeatedly prior to or after SBT, it was registered once in each group to provide a concise overview over the applied treatment modalities

SBT, stereotactic brachytherapy; EBRT, external beam radiotherapy

The clinical and radiological follow-up took place for all patients in compliance with a standardized recall protocol. The first examination was carried out three months after SBT and semi-annually thereafter, until malignant progression (thereafter three-months intervals), death or last follow-up [13]*.* To verify the time and cause of death at last follow-up, information was matched with registration offices and the provincial Cancer Registry. During the final database update, patients alive were either seen at an appointment in our clinic or called by phone. 13 patients are lost to follow-up (4 patients are from abroad, new address was unknown in 4 patients and 5 patients were not reachable by phone during the database update period).

Clinical exacerbation that indicated tumor recurrence/progression or malignant transformation, had to be confirmed by neuroradiological diagnostic and—in unclear cases—by histological re-evaluation of tumor samples obtained by stereotactic biopsy or open surgery if indicated.

### Treatment planning, implantation techniques and dosimetry

The stereotactic procedures of biopsy and seed-implantation have been published in various scientific articles by Kreth et al. [8, 14–17].

Before seed-implantation can be performed, a histological confirmation of a LGGII is stipulated. Preferentially one week prior to SBT, serial biopsies were obtained using a computed tomography (CT) guided stereotactic device. Occasionally, histology was obtained by anteceding tumor surgery (see above). Thereafter, the cases were presented to the interdisciplinary tumor board. The boards’ recommendation to perform SBT was discussed with the patient and an informed consent was obtained.

**Fig. 1:**
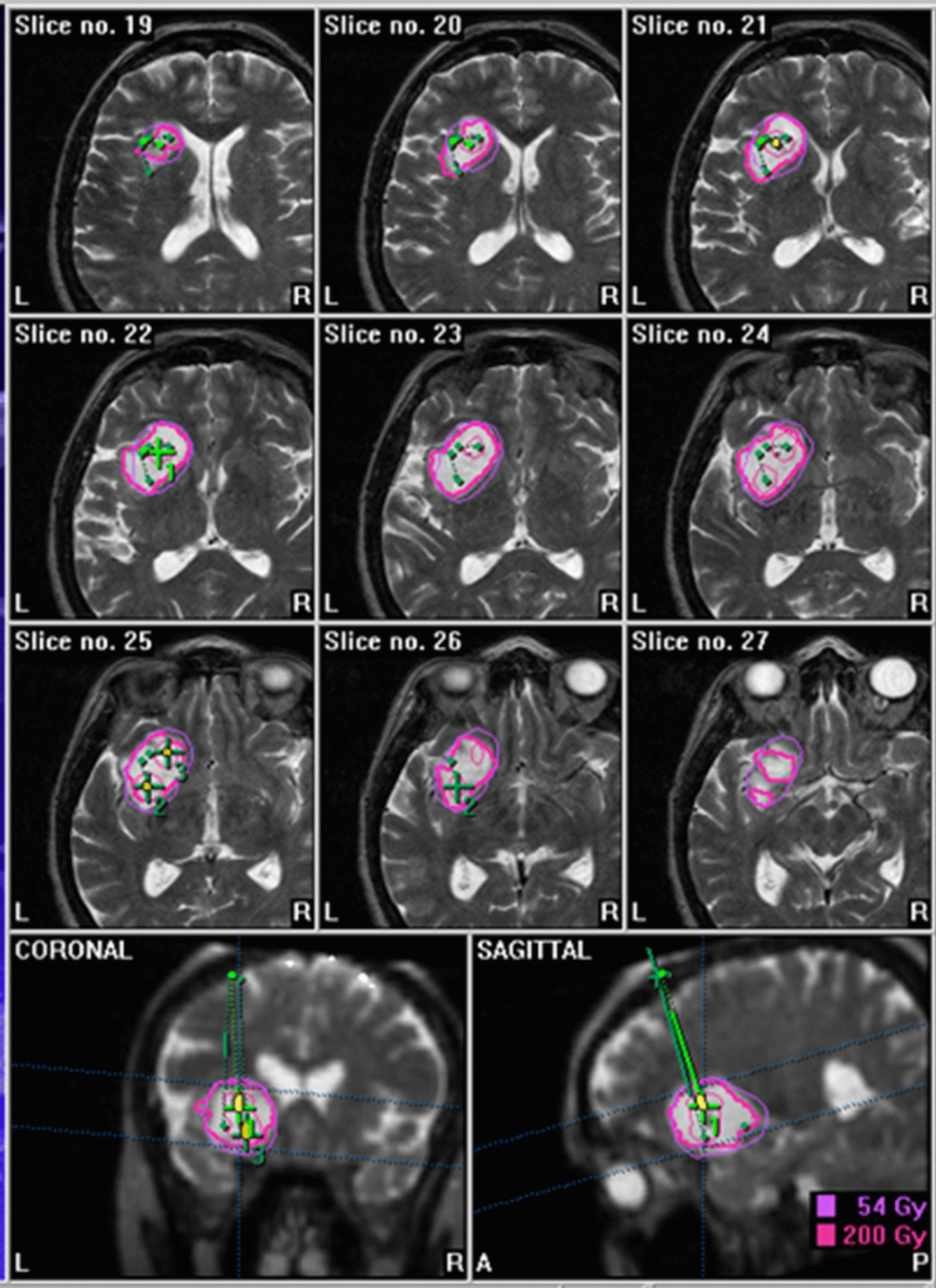
Example for a treatment plan in a patient with grade II astrocytoma (seeds 3, volume 14.4 ml, reference dose 54 Gy, minimum dose 36 Gy, maximum dose 763 Gy, total implanted activity 31.7 mCi)

The ideal number of seeds, localization, and trajectory, as well as the prescribed dose was defined with a target software program (Brainlab AG, Target software, version 1.19, Feldkirchen, Germany). To compute the three-dimensional treatment plan and receive an accurate volume calculation and isodose distribution, a fusion of the stereotactically localized CT with a preoperative magnetic resonance imaging (MRI) is required (Fig. 1). It was ascertained that blood vessels were not in the high dose area (≥ 200 Gy) and that radiosensitive structures (optical nerve, chiasm, mammillary bodies, etc.) were preserved [18, 19]. The Iodine-125 seeds (Theragenics Corporation, Bufford, GA, USA) (measuring 4.5 × 0.8 mm), loaded into Teflon catheters, were then stereotactically inserted via 2 mm burr-holes for each catheter in the skull. Within 24 h after surgery a CT was performed and compared with the preoperative scan to verify the proper position of the temporary implant. Dexamethasone was administered on the day of the procedure and tapered over the next 3 days. After a median irradiation time of 24.8 days (range 10–50) the seed catheters were explanted.

The average length of hospitalization amounted to about 3 days.

A detailed description of the SBT parameters is given in Table 1.

### Statistical analysis

The reference point for this study was the date of first diagnosis or first seed-implantation. The time interval between first diagnosis and last follow-up was used for estimation of overall survival (OS) after first diagnosis. The time between SBT and last follow-up, was termed OS after SBT.

Progression-free survival (PFS) after seed-implantation was evaluated according to the time of last follow-up or the time of tumor recurrence/progression.

Kaplan–Meier-Analysis was performed. Mean survival was estimated as the integral of the untruncated Kaplan–Meier survival curve, and median survival, 5-year survival and 10-year survival were directly read off the Kaplan–Meier survival curve. Survival-related significance was tested by the Mantel-Cox test (log-rank test) and *p-*values were calculated from the appropriate χ^2^-statistic. Other associations between continuous quantities were evaluated for significance with the two-sided unpaired t-test, and associations between discrete properties were evaluated for significance with the two-tailed Fisher’s exact test.

## Results

### Patients demographical and clinical characteristics

Overall, the study included WHO grade II astrocytomas (n = 90), oligoastrocytomas (n = 8) or oligodendrogliomas (n = 8). Table 1 summarizes the patients’ characteristics, histopathology, and molecular biomarkers for all patients and the group of patients who had SBT as first-line therapy. Table 3 displays a detailed overview of the different treatment modalities before reference SBT and salvage therapy after reference SBT of this analysis.

### Survival data and treatment response

#### Follow-up

The series comprised a median follow-up period after first diagnosis of 115.9 months (range 1.6–405.5) and of 86.7 months (range 1.1–222.3) after SBT. At last follow-up, 48 patients were alive and 58 had died. The leading cause of death was malignization in 35 patients and unrestrained tumor growth in 17 patients. 28 of the 106 patients were free of progression until last follow-up (median follow-up of 104 months for this subpopulation; range 1.1–222.3).

#### Overall survival and progression-free survival

The median duration between diagnosis and first seed-implantation amounted to 4.2 months (range 0–248).

The mean OS from first diagnosis was 186 months. Median OS after first diagnosis was 169 months. Five- and ten-years survival rates after first diagnosis were 79% and 62%, respectively, with no significant difference between astrocytomas and oligodendrogliomas/oligoastrocytomas. No flattening of the Kaplan–Meier curve was observed over time.

The mean and median OS after SBT resulted in 125 and 108 months, respectively. Five-years survival rate after SBT was 72% (95% CI 64–81) and 10-years survival rate after SBT averaged out to 43% (95% CI 32–53).

**Fig. 2:**
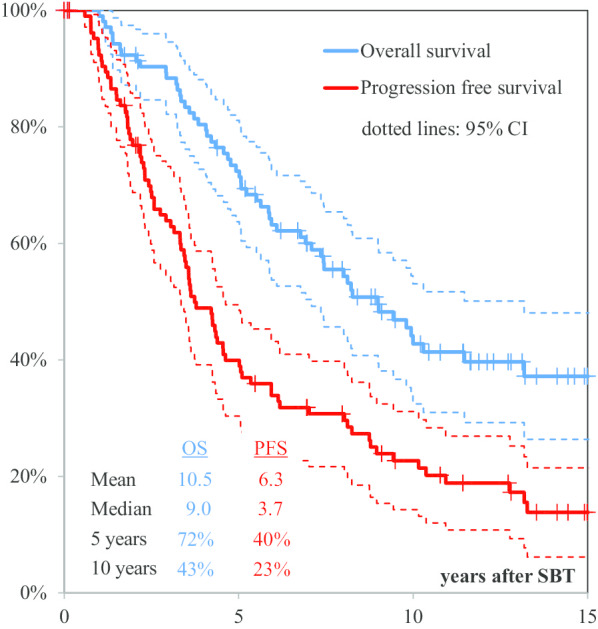
Overall survival (OS) and progression-free survival (PFS) after stereotactic brachytherapy (SBT) of all 106 patients

The mean and median PFS duration after SBT were 76 and 45 months, respectively. Five- and ten-years PFS rates after SBT were 40% (95% CI 30–49) and 23% (95% CI 14–31), respectively (Fig. 2).

Tumor recurrence/progression was confirmed in a total of 75 patients and 7 presented with tumor progression within less than twelve months after SBT. Those seven patients were on average 51.8 years old at first SBT. Tumor malignization was histologically verified in five out of these seven patients, after a mean of 13.7 months.

#### SBT as first-line therapy

The mean and median OS and 5- and 10-years survival rates after first diagnosis for the patients that received SBT as initial treatment were 131 and 124 months, 71%, and 56%, respectively. The mean and median OS after SBT averaged out to 129 and 138 months and the 5- and 10-years survival rates were 71% (95% CI 58–84), and 54% (95% CI 39–68), respectively. Analyzing mean and median PFS after SBT under the same aspects, 87 and 55 months, 45% (95% CI 31–59), and 33% (95% CI 19–47) were obtained, respectively (*p* = 0.1).

### Prognostic factors for post-SBT-survival

Prognostic factors for post-SBT-survival are summarized in Table 4.

**Table 4:** Prognostic factors for overall survival (OS) and progression-free survival (PFS) after stereotactic brachytherapy (SBT)

Prognostic factor	No	% 5-/10-years OS	% 5-/10-years PFS	*p-*value OS/PFS
**Sex**				0.45/0.50
Female	47	85/59	52/35	
Male	59	71/50	40/26	
**Age at first SBT**				0.68/0.55
< 40 years	50	79/59	46/25	
> 40 years	56	75/50	44/34	
**Tumor volume at SBT in ml**				0.15/0.44
< 10 ml	55	83/63	53/41	
> 10 ml	56	66/30	26/14	
**Histology**				0.75/0.06
Astrocytoma	90	76/54	47/31	
Oligoastrocytoma/Oligodendroglioma	16	79/57	28/–	
**MGMT methylation**				0.73/0.80
Yes	42	87/53	37/–	
No	9	75/–	50/–	
**LOH 1p/19q**				0.27/0.82
Yes (= loss of either or both)	20	94/74	52/–	
No	27	76/40	37/20	

OS, overall survival; PFS, progression-free survival; No, number; SBT, stereotactic brachytherapy; LOH, loss of heterozygosity; MGMT methylation, o-6methylguanine-DNA methyltransferase methylation

#### Age, gender and tumor volume

Age (< > 40 years), gender and tumor volume did not have a significant influence on either OS or PFS after SBT.

#### MGMT and LOH status, and malignant transformation

MGMT methylation status and LOH 1p/19q status had no influence on PFS and OS after SBT.

Malignant transformation was registered in 46 cases. The median age at the time of malignization was 45.2 years (range 18.8–72.5). Malignancy occurred as soon as 9.1 months after first seed implantation and the latest manifested 197 months after first seed implantation. The median time to malignization after first seed implantation was 52.8 months. The 5- and 10-years malignant transformation rates in our patient population after first seed implantation were 25%, and 39%, respectively.

To determine risk factors for malignization, the following variables were investigated: gender (*p* = 0.76), age (*p* = 0.33 for older/younger than 40 years at SBT), tumor volume (*p* = 0.052 for larger/smaller than 10 ccm) and histology (*p* = 0.81 for oligo vs. non-oligo).

### Perioperative mortality and morbidity

The procedure-related mortality rate was zero, although an overall complication rate of 16% was registered. 89 of 106 patients showed no signs of complications until last follow-up and 17 developed side-effects potentially associated with seed-implantation.

Treatment-induced complications are summarized in Table 2. As for periprocedural complications, two patients suffered from cerebral bleeding and in three patients seed repositioning became necessary due to migration and/or positional deviation from the original treatment plan. A single case was registered for wound healing disturbance with concomitant cerebritis whereupon a premature seed-explantation had to be performed. Long-term complications included 11 prolonged edemas in varying degrees of severity (mostly grade 1 and 2) which could be attributed to radiation. Three of those patients presented with radionecrosis: One female patient developed a severe cranial pressure symptomatology with hemiparesis three months after seed implantation which required high dose dexamethasone therapy for several months. In another female patient radionecrosis with brainstem compression was observed. After long-term dexamethasone therapy reduction failed and the patient was advised to have radionecrosectomy 1 year after seed implantation. Radionecrosectomy was also necessary in one male patient treated in 2000 with a treatment dose of 60 Gy who developed a severe edema 1 year after seed implantation. The other eight patients (four females, four males) suffered from edemas causing headaches. They occurred one to eight months after seed implantation, required dexamethasone therapy, and were completely regressive four to twelve months after seed implantation.

Median tumor volume was 16.9 ml in patients with radiogenic complications, but only 7.7 ml in patients without complications (*p* = 0.029).

The complication rate was not influenced by number of implanted seeds or delivered reference dose. Brain necrosis coincided in patients with a median tumor volume of 8.4, 16.6, and 21.5 ml.

The occurrence of complications correlated with a worse prognosis for survival after SBT (median OS 5.6 years vs. 9.8 years, *p* = 0.039).

## Discussion

The 5- and 10-years OS after first diagnosis and after SBT of our long-term analysis of 79% and 62%, and 72% and 43%, respectively, confirmed those of other studies on SBT in low-grade gliomas [11]. Mehrkens et al., for example, reported in 2004 a 5-years OS rate of 55% in their subset of patients [9], Kreth et al. found in 2006 5- and 10-years OS rates of 56% and 37%, respectively, for their cohort [13], and Schnell et al. registered in 2008 a 5-years OS of 93% in a selected population [10].

The results of our SBT series also compare well to the outcomes of another high precision radiotherapy mode, stereotactic radiosurgery. With this technique, 5- and 10-years OS rates of 76–89% and 65–74% have been reported [5]. Both treatment modalities are recommended now for selected patients in the evidence-based clinical practice guidelines [5]. However, radiobiology is very different in SBT and stereotactic radiosurgery. While SBT is a protracted dose application, during stereotactic radiotherapy doses are delivered at a high dose rate. Shrieve et al. directly compared SBT and stereotactic radiosurgery in patients with recurrent glioblastoma and found similar survival rates with both modalities [20]. Nevertheless, these results were obtained from high-grade gliomas and are only transferable to LGG to a limited extent.

Our analysis shows similar results to a prospective trial on proton therapy for LGGII, which requires 6 weeks of treatment time [21]. The dosimetric advantages of proton therapy over conventional radiotherapy have been demonstrated in another analysis, especially in organs at risk sparing [22]. With SBT, with its steep dose gradients, organs at risk sparing is guaranteed as well.

In the meantime, it has been shown that the addition of chemotherapy (procarbazine, CCNU, and vincristine) to radiotherapy can improve survival in patients younger than 40 years of age with subtotal resection and in all patients over 40 years of age. Ten-years PFS could be improved from 21 to 51%, and 10-years OS from 40 to 60% [23, 24]. It has not so far been prospectively studied whether survival rates in LGG patients could be further improved through an early treatment combination of SBT and chemotherapy.

Ten-years OS and PFS rates as high as 89.8% and 47.3% have been reported for a combination treatment of SBT and EBRT in oligodendroglial brain tumors WHO grade II [25]. In our patient cohort only 16 specimens were classified as oligoastrocytoma or oligodendroglioma, known to be favorable histologies. Interestingly, histology and LOH 1p/19q were no prognostic factors for OS and PFS in our analysis.

A complication rate of 16% seems high initially. However, it is an overall complication rate that includes not only severe, but all complications and covers not only the periprocedural time, but the entire follow-up period. Severe complications (radionecrosis, local cerebral bleeding, cerebritis) occurred in 6% of cases, which is in line with other brachytherapy studies [11, 25]. Comparison with other radiotherapy methods (e.g. radiosurgery) is challenging, but toxicity seems to be similar [5]. Comparison with surgical complication rates is even more challenging as different techniques (e.g. awake surgery) are used and our study also included tumors in eloquent locations harboring high risks for surgical complications.

These promising results of SBT in low-grade gliomas, in contrast to the disappointing studies of high-grade glioma brachytherapy, may partly derive from target volume definition and to -some extent- selection bias. In our study only well circumscribed low-grade gliomas < 4 cm have been implanted. High-grade gliomas are much more infiltrating and thus not very well suited for a highly focused radiotherapy technique like brachytherapy or radiosurgery [26]. As tumor volume is a negative predictor for survival [15, 27, 28], the good survival rates may have been biased by the small and circumscribed tumor features that were required for SBT [13, 29, 30]. The prognostic scoring system formulated by the European Organization for Research Treatment of Cancer Brain Tumor Cooperative Group and Radiotherapy Cooperative Group, found that in those age ≥ 40 years, astrocytoma histology and largest tumor diameter ≥ 6 cm are unfavorable prognostic factors [31]. The analysis of our patient cohort did not reveal larger tumor volume as negative prognostic factor (though, largest implanted volume was 50.5 ml), and neither astrocytoma histology nor age influenced OS. Again, this stresses the selection bias caused by the maximum implantable tumor volume of SBT, which may have confounded the outcome. On the other hand, tumors in vicinity of eloquent brain areas deemed inaccessible to safe tumor resection have been included in this study, as well. In lesions that are feasible for microsurgical treatment, complete tumor resection is still regarded as the gold standard in LGGII [32–35]. For eloquently located LGG Schnell et al. have shown that an incomplete but safe surgical resection in combination with SBT is a good option [12]. In our patient cohort 19 patients had a planned partial resection before SBT of the residual disease. Whenever tumor resection is not safe in even small lesions but eloquent brain areas SBT is a minimally invasive, safe alternative with good therapeutic effects.

Beside the mentioned selection bias, another limitation of our analysis is the lack of molecular markers in a large proportion of our patients. The patients of the study were treated between 1997 and 2011 but molecular marker analyses were not introduced until 2004 for LOH, until 2007 for MGMT and until 2008 for IDH. Therefore, the status is unknown in 56%, 52% and 75% of patients, respectively. In recent years, it has become more and more evident that the heterogeneous outcome in patients with low-grade gliomas may be explained in large proportion by molecular markers. Therefore, IDH, LOH and MGMT status are currently included not only in classification but also in prognosis determination and treatment planning [2, 36–38].

## Conclusion

Our analysis further confirmed that SBT is a safe and minimally invasive treatment modality with good outcome in selected patients, which can postpone the application of secondary treatment.

It is an alternative primary treatment method as well as an adjunct to open tumor resection, with acceptable complication rates. SBT can expand the options of interdisciplinary and individualized multimodal treatment of LGGII.

## Data Availability

The datasets used and analysed during the current study are available from the corresponding author on reasonable request.
